# Global temperature change potential of nitrogen use in agriculture: A 50-year assessment

**DOI:** 10.1038/srep44928

**Published:** 2017-03-21

**Authors:** R. K. Fagodiya, H. Pathak, A. Kumar, A. Bhatia, N. Jain

**Affiliations:** 1 Centre for Environment Science and Climate Resilient Agriculture, ICAR-Indian Agricultural Research Institute, New Delhi 110012, India

## Abstract

Nitrogen (N) use in agriculture substantially alters global N cycle with the short- and long-term effects on global warming and climate change. It increases emission of nitrous oxide, which contributes 6.2%, while carbon dioxide and methane contribute 76% and 16%, respectively of the global warming. However, N causes cooling due to emission of NO_x_, which alters concentrations of tropospheric ozone and methane. NO_x_ and NH_3_ also form aerosols with considerable cooling effects. We studied global temperature change potential (GTP) of N use in agriculture. The GTP due to N_2_O was 396.67 and 1168.32 Tg CO_2_e on a 20-year (GTP_20_) and 439.94 and 1295.78 Tg CO_2_e on 100-year scale (GTP_100_) during years 1961 and 2010, respectively. Cooling effects due to N use were 92.14 and 271.39 Tg CO_2_e (GTP_20_) and 15.21 and 44.80 Tg CO_2_e (GTP_100_) during 1961 and 2010, respectively. Net GTP_20_ was 369.44 and 1088.15 Tg CO_2_e and net GTP_100_ was 429.17 and 1264.06 Tg CO_2_e during 1961 and 2010, respectively. Thus net GTP_20_ is lower by 6.9% and GTP_100_ by 2.4% compared to the GTP considering N_2_O emission alone. The study shows that both warming and cooling effects should be considered to estimate the GTP of N use.

Nitrogen is the most limiting nutrient controlling the primary production of agricultural systems. Intensively cultivated systems require external application of N to increase and sustain global food production. Consumption of fertilizer N has increased globally from ~12 Tg in 1960 to ~113 Tg in 2010^1^. If current N consumption trends continues, considerably higher amount of fertilizer N will be used in agriculture to provide food for an additional 2 billion people by 2050^2^. The N cycle involves five steps i.e., N fixation (N_2_ → NH_3_/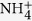), nitrification (NH_3_/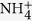 → 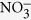), assimilation (uptake of 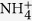 and 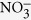 into plant tissues), ammonification (organic N → NH_3_) and denitrification (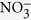 → N_2_)^3^. During the N cycle several reduced (NH_3_) and oxidised N compounds (NO_x_, NO, N_2_O, 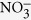) are emitted to the atmosphere affecting the climate system^4^.

Climate change due to emission of greenhouse gases (GHGs) viz. carbon dioxide (CO_2_), methane (CH_4_) and nitrous oxide (N_2_O) contributing 76.0%, 16.0% and 6.2%, respectively is likely to affect agricultural productivity and food security adversely^5^. Addition of N in agricultural soil alters the fluxes of GHGs^6^,^7^,^8^. The reactive N (Nr) has direct as well as indirect effects on N_2_O emission from agricultural soil^9^,^10^,^11^,^12^. Emission of N_2_O is a major concern because of its long atmospheric lifetime (about 116 years), higher global warming potential (GWP) i.e., 310 times that of CO_2_^5^ and high global temperature change potential (GTP) of 290 on 100-year basis^13^.

The GWP is the global mean radiative forcing of 1 kg pulse emissions of a greenhouse gas relative to 1 kg of reference gas i.e., CO_2_^14^. The GWP is an index of time-integrated radiative forcing. However, it does not give a quantitative information on effect of GHG emission on global temperature^13^,^15^,^16^. The GTP is the global average temperature change at time t due to emission of a GHG relative to CO_2_ emission^13^,^17^. The GTP is directly related to surface temperature changes as a result of GHG emission. Thus GTP has an advantage in quantifying temperature change compared to GWP.

In addition to N_2_O emission, N use in agriculture results in increased emission of NH_3_ and NO_x_ contributing to climate change indirectly^18^. The NO_x_ impacts global warming by (i) formation of ozone (O_3_), which contributes to warming^19^ and (ii) removal of CH_4_ by hydroxyl radical, thus contributing to cooling^20^. Moreover, CH_4_ enhances ozone formation in the upper atmosphere over longer time-scales. Thus NO_x_ can also reduce production of O_3_ and contribute to cooling^21^. Both NO_x_ and NH_3_ enhance formation of light-scattering sulphate and organic aerosols. NO_x_ can be oxidised to form nitric acid (HNO_3_), which forms aerosols of ammonium nitrate (NH_4_NO_3_) in presence of NH_3_^22^. Moreover, use of N usually increases net primary productivity with more CO_2_ fixation in terrestrial systems^23^,^24^,^25^,^26^ and enhances carbon sequestration in soil due to more litter production^27^. The direct and indirect impacts of reactive N (Nr) on global warming and cooling are summarized in Table 1.

The previous reports have evaluated the emission of N_2_O only due to N use in agriculture for a short period. However, besides global warming due to N_2_O emission, N use in agriculture has other direct and indirect effects causing warming and cooling. To assess the impacts of N use on climate change, therefore, the warming as well as cooling effects should be considered^18^. Moreover, such warming and cooling effects need to be assessed for a sufficiently long period as the N use in global agriculture has undergone substantial changes in the last decades. The present study quantified the global warming and cooling potentials of N use in global agriculture during last 50 years (1961–2010).

## Results and Discussion

### Total N input in global agriculture

**Figure 1:**
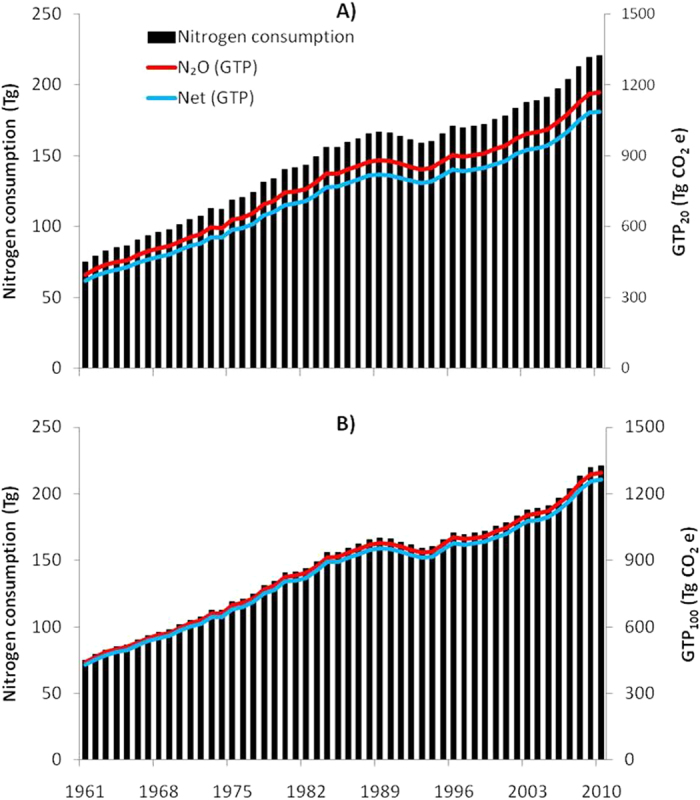
Total nitrogen consumption, global temperature change potential (GTP) due to N_2_O emission alone and net GTP of N use in global agriculture (**A**) 20-year and (**B**) 100-year time-scales.

Total N input in global agriculture increased by 2.95 times during 1961 to 2010 (Fig. 1 and Table 2). In 1961, total N input from different sources was 74.93 Tg N. Animal manure accounted the highest amount (32.30%), followed by biological N fixation (BNF, 29.33%), crop residues (18.75%), fertilizer N (15.47%) and atmospheric deposition (4.16%) (Table 2). In 2010, total N input was 270.70 Tg N (Fig. 1 and Table 2) and fertilizer N was the largest source (51.38%) followed by animal manure (15.41%), crop residue (14.40%), BNF (12.31%) and atmospheric deposition (6.49%) (Table 2).

### GTP of N_2_O emission

**Figure 2:**
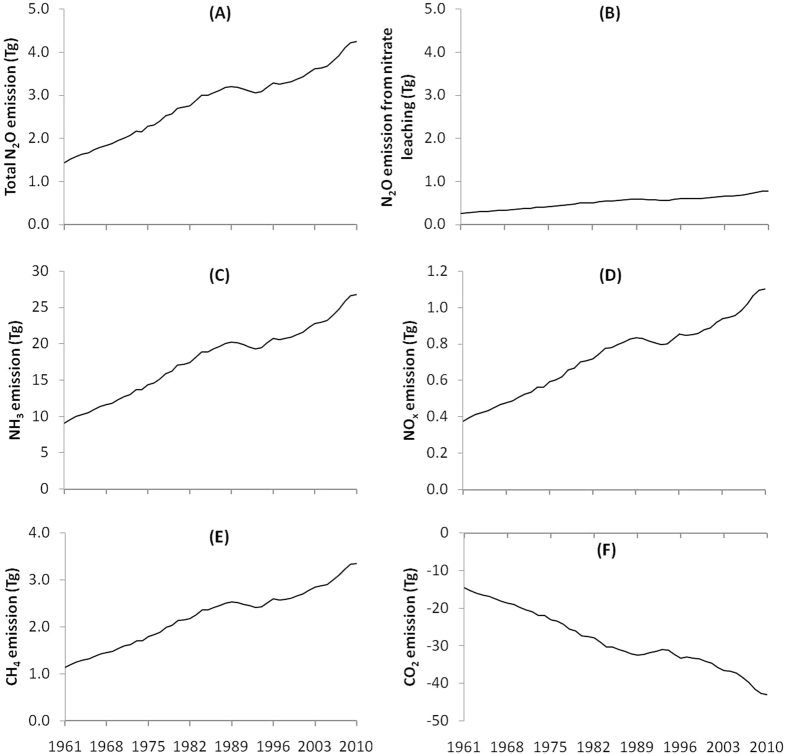
Emissions of total (direct + indirect) N_2_O (**A**), N_2_O from nitrate leaching (**B**), NH_3_ (**C**), NO_X_ (**D**), CH_4_ (**E**) and CO_2_ (**F**) from global N use in agriculture during 1961–2010.

**Figure 3:**
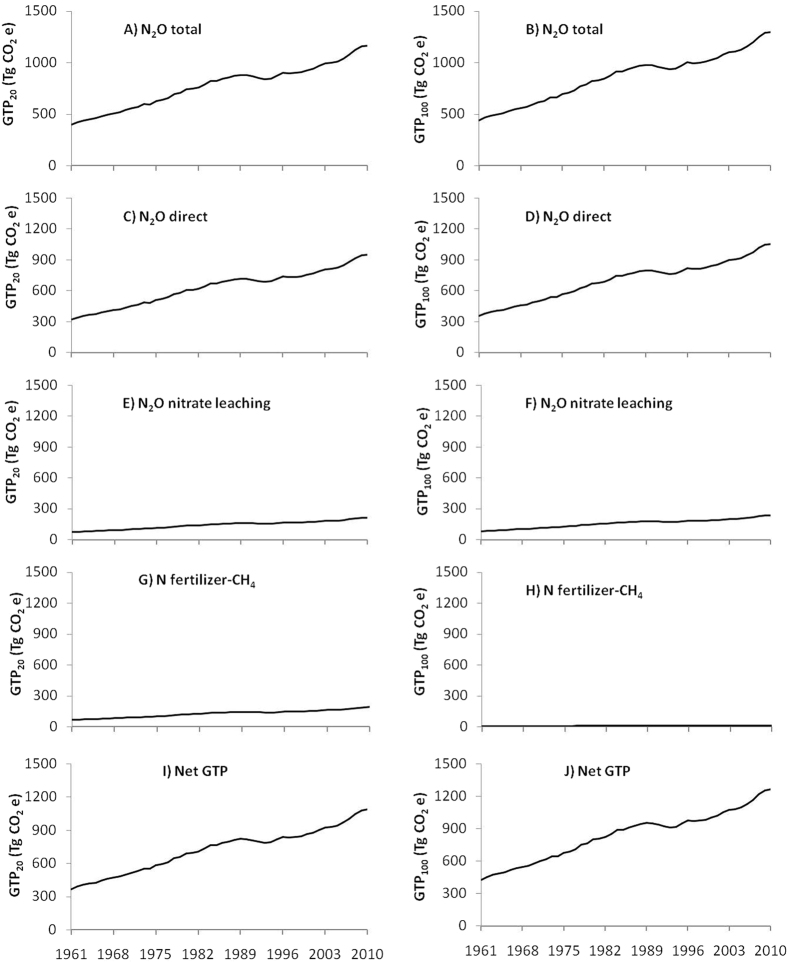
Warming or global temperature change potential (GTP) due to total (direct + indirect) N_2_O emission (**A**,**B**), direct N_2_O emission including atmospheric deposition (**C**,**D**), N_2_O emission from nitrate leaching (**E**,**F**), N fertilizer and CH_4_ flux (**G**,**H**) and net GTP (**I**,**J**) of global N use in agriculture on 20-year (left) and 100-year (right) times-scales.

Total N_2_O emission from agriculture increased from 1.44 Tg to 4.25 Tg during 1961 to 2010 (Fig. 2A). The GTP of total N_2_O emission, thus increased from 396.67 to 1168.32 TgCO_2_e in a 20-year time-scale (GTP_20_) (Fig. 3A) and from 439.94 to 1295.78 Tg CO_2_e in 100-year time-scale (GTP_100_) (Fig. 3B) during 1961 to 2010.

### GTP of NH_3_ and NO_x_ emissions

**Figure 4:**
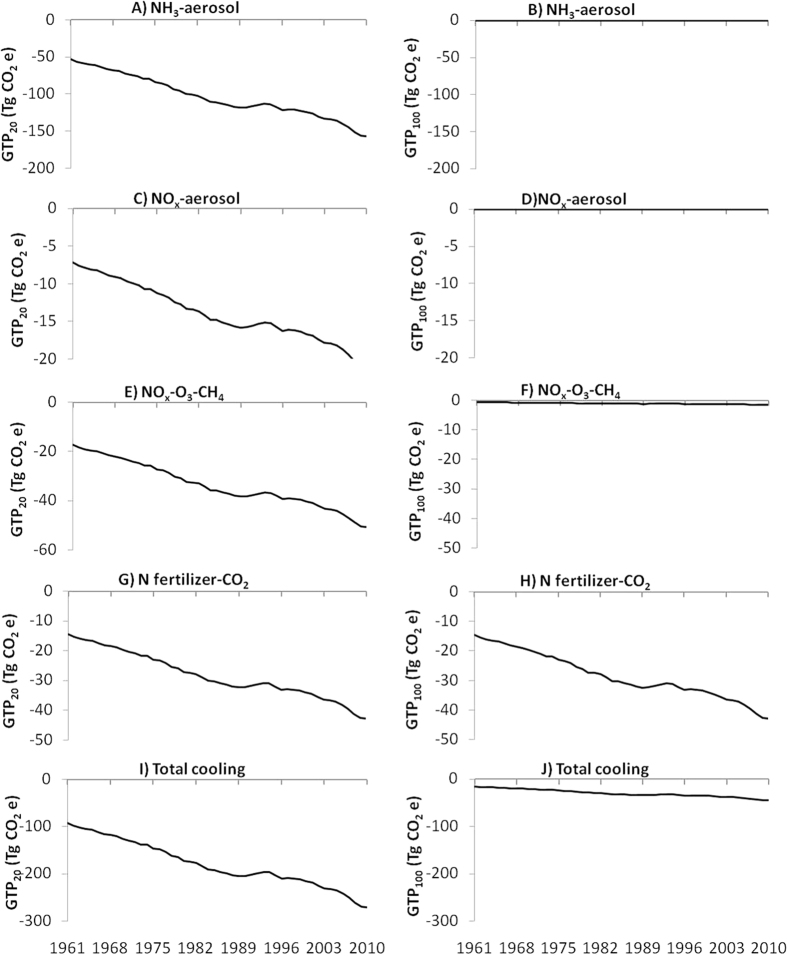
Cooling or Global temperature change potential (GTP) due to NH_3_ aerosol (**A**,**B**), NO_x_ aerosol (**C**,**D**), NO_x_-O_3_-CH_4_ (**E**,**F**), CO_2_ with N fertilizer (**G**,**H**) and total cooling (**I**,**J**) of global N use in agriculture on 20-year (left) and 100-year (right) times-scales.

Emission of NH_3_ from global agriculture was 9.10 and 26.80 Tg during 1961 and 2010, respectively (Fig. 2C). Emission of NO_x_ was 0.37 and 1.10 Tg during 1961 and 2010, respectively (Fig. 2D). Cooling impacts due to these emissions of NO_x_ and NH_3_ were 77.58 and 228.50 Tg CO_2_e in GTP_20_ and 0.65 and 1.91 Tg CO_2_e in GTP_100_ during 1961 and 2010, respectively (Fig. 4). Aerosol formation from NH_3_ contributed 69% of the cooling effect, followed by ozone and CH_4_ alternation due to NO_x_ (22%) and aerosol formation from NO_x_ (9%) (Fig. 4A,C,E). However, on GTP_100_ (Fig. 4B,D,F) these cooling impacts of NH_3_ and NO_x_ were smaller compared to GTP_20_ indicating that as the time horizon becomes longer, short-lived compounds have less effects on GTP^18^.

### GTP due to altered CH_4_ and CO_2_ fluxes

The CH_4_ is produced in soil during microbial decomposition of organic matter under anaerobic conditions. Soils submerged under water, rice fields for example, are the potential sources of CH_4_. Addition of N increases CH_4_ emission by inhibiting CH_4_ oxidation and reducing CH_4_ uptake in aerobic soils due to increased concentration of ammonium (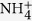)^28^ and nitrate (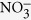)^29^,^30^ in soil. This increase in CH_4_ flux due to N use in agriculture ranged from 1.14 Tg in 1961 to 3.35 Tg in 2010 (Fig. 2E) contributing to 42.14 and 124.12 Tg CO_2_e in GTP_20_ (Fig. 3G) and 4.44 and 13.80 Tg CO_2_e in GTP_100_ (Fig. 3H) in 1961 and 2010, respectively. Fluxes of CO_2_ decreased by14.56 Tg to 42.89 Tg during the same period (Fig. 2F) due to increased uptake of CO_2_ as a result of N application (Fig. 4G,H).

### Net impact of N use in agriculture on GTP

Net GTP of N use in agriculture was 369.44 and 1088.55 Tg CO_2_e on GTP_20_ (Fig. 1A) and 429.17 and 1264.06 Tg CO_2_e on GTP_100_ (Fig. 1B) in 1961 and 2010, respectively. The net GTP_20_ was lower by 6.9% and GTP_100_ by 2.4% compared to the respective GTPs when N_2_O emission alone was considered.

### Total GTP during 1961–2010

**Figure 5:**
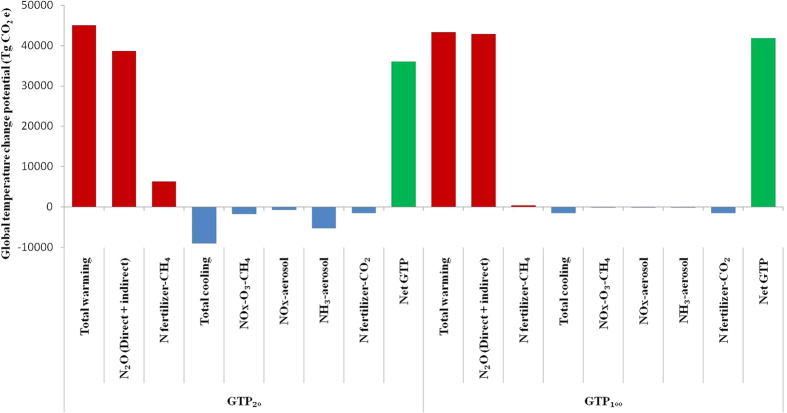
Total Global temperature change potentials of global N use in agriculture in 50 year on 20-year (left) and 100-year (right) times-scales.

Total warming due to N use in global agriculture during 50 years was 45041.92 Tg CO_2_e in GTP_20_ and 43362.98 Tg CO_2_e in GTP_100_ (Fig. 5). Emission of N_2_O due to N use in agriculture contributed 86% and 99% of this warming in GTP_20_ and GTP_100_, whereas CH_4_ contributed 14% and 1% in GTP_20_ and GTP_100_, respectively. Total cooling was 8991.28 and 1484.19 Tg CO_2_e in GTP_20_ and GTP_100_, respectively (Fig. 5). The major cooling was due to NH_3_ aerosol formation (57.8%) followed by NOx induced O_3_ and CH_4_ alteration (18.7%), N fertilizer-induced C sequestration (15.8%) and NO_x_ aerosol (7.7%). However, on GTP_100_ N fertilizer-induced C sequestration contributed the maximum (95.74%) and others were marginal.

The net GTP_20_ was 36050.64 Tg CO_2_e i.e., 6.84% lower and GTP_100_ was 41878.79 Tg CO_2_e i.e., 2.45% lower compared to the respective GTPs when warming due to N_2_O emission alone was considered.

## Methods

### Total N use in global agriculture

Total N input in global agriculture (N_T_) was calculated using the equation (1).


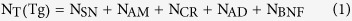


Where, N_SN,_ N_AM_, N_CR,_ N_AD_ and N_BNF_ are amounts of N added (Tg) to soil annually through fertilizer, animal manure, crop residue, atmospheric deposition, and biological nitrogen fixation (BNF), respectively. Data on N_SN,_ N_AM_, N_CR_ were obtained from FAOSTAT^1^. The N_AD_ and N_BNF_ were calculated as per the equations (2) and (3) respectively.


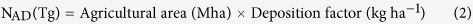



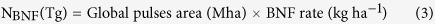


Data on area under global agricultural and pulse crops were obtained from FAOSTAT^1^ whereas data on deposition factor were calculated from Liu *et al*.^31^ and Liu *et al*.^32^ and BNF were calculated from Liu *et al*.^31^.

### Emission/uptake factors

Emission and uptake factors (EF) used in the study are mentioned in Table 3. Factor for direct N_2_O emission was taken as 0.01^33^ and N_2_O from 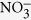 leaching was 0.0075^33^. Emission factor for 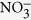 leaching, NH_3_ and NOx emissions were 0.3^33^, 0.10^33^ and 0.005^34^ kg kg^−1^ N applied, respectively. Emissions of CH_4_ from anaerobic and aerobic fields were taken as 0.008 and −0.012 kg CH_4_-C ha^−1^ yr^−1^ kg^−1^ N aplied^24^. The factor for C sequestration was 0.053 kg CO_2_-C ha^−1^ yr^−1^ kg^−1^ N^24^.

### Emission/uptake fluxes

Total flux (F_T_) of N_2_O, 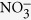 leaching, NO_X_, NH_3_, CH_4_ and CO_2_ were calculated using the equation (4).


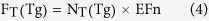


Where N_T_, is total amount of N (Tg) added to agricultural land and EFn is the respective emission/uptake factor.

N_2_O flux from 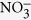 leaching was calculated using the equation (5).


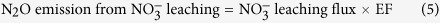


### GTP of N_2_O, NO_X_, NH_3_, CH_4_ and CO_2_ fluxes

The GTP of N_2_O, NO_x_ and NH_3_ fluxes were calculated using the equation (6).


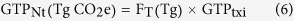


Where GTP_Nt_ is GTP at ‘t’ time-scale i.e., 20 or 100 years; F_T_ is flux of NO_x_, NH_3_ and N_2_O emission (kg yr^−1^), GTP_txi_ is GTP for ‘i’ kg of ‘x’ compound (N_2_O, NOx, NH_3_) at time-scale ‘t’. GTP_20_ and GTP_100_ used in the study are mentioned in Table 4.

The following equation (7) was used to calculate GTP of CH_4_ and CO_2_ emission/uptake (GTPCt).


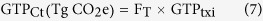


Where GTP_txi_ is GTP for ‘i’ kg of ‘x’ compound (CH_4_ and CO_2_) at time-scale ‘t’.

Finally, the net GTP (GTP_T_) of N addition to global agriculture was calculated using the equation (8).


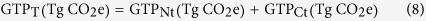


## Summary

Globally, nitrogen is the most widely used nutrient in agriculture. Nitrogen fertilizer acts as a source of global warming as it contributes to N_2_O emission. However, it also contributes to global cooling with emissions of NH_3_ and NO_x_. Therefore, while assessing global temperature change potential (GTP), both the warming and cooling effects of N use in agriculture should be considered. Our estimates showed that net GTP in 20-year time-scale is 6.9% lower and in 100-year time-scale 2.4% lower when warming as well as cooling effects of N use in agriculture were considered compared to considering warming due to N_2_O emission alone.

## Additional Information

**How to cite this article:** Fagodiya, R. K. *et al*. Global temperature change potential of nitrogen use in agriculture: A 50-year assessment. *Sci. Rep.* **7**, 44928; doi: 10.1038/srep44928 (2017).

**Publisher's note:** Springer Nature remains neutral with regard to jurisdictional claims in published maps and institutional affiliations.

## Figures and Tables

**Table 1:** Gaseous emission process altered by reactive nitrogen, climate forcing elements, process of warming/cooling and their overall impacts.

Gaseous emission process altered by reactive N	Climate forcing element	Process of warming/cooling	Overall impacts
1. N_2_O	N_2_O	Emitted from agricultural soils	Warming
2. NOx → ozone and CH_4_	Ozone, CH_4_	NOx perturbs the chemical production and destruction of the greenhouse gases ozone and CH_4_.	Cooling
3. NOx → aerosol	Nitrate, ammonium aerosol	NOx can enhance the formation of light-scattering aerosols.	Cooling
4. NH_3_ → aerosol	Nitrate, ammonium aerosol	NH_3_ enhances the formation of light-scattering aerosols.	Cooling
5. N fertilizer → CO_2_ flux	CO_2_	On croplands, nitrogen from fertilizer and manure may enhance the storage of CO_2._	Cooling
6. N fertilizer → CH_4_ flux	CH_4_	On croplands, N from fertilizer and manure may perturb uptake and emission of CH_4_.	Warming

Source: Modified from Pinder *et al*.^18^.

**Table 2:** Sources of nitrogen and their contribution in global agriculture.

Sources of N	Nitrogen (Tg)	
	1961	2010
Fertilizer	11.59 (15.47)^a^	113.40 (51.38)
Animal manure	24.20 (32.30)	34.02 (15.41)
Crop residue	14.05 (18.75)	31.79 (14.40)
Atmospheric deposition	3.12 (4.16)	14.33 (6.49)
Biological N fixation	21.98 (29.33)	27.16 (12.31)
Total	74.93 (100)	220.70 (100)

Source: FAOSTAT^1^

^a^Figures in the parenthesis are percent of total N.

**Table 3:** Emission and uptake factors of different parameters used in the present study.

Sl. No.	Parameters	Emission/uptake factor	Unit	Source
1	Direct N_2_O-N	0.01	kg N_2_O-N ha^−1^ yr^−1^ kg^−1^N	33
2	N_2_O-N from nitrate leaching	0.0075	kg N_2_O-N ha^−1^ yr^−1^ kg^−1^N	33
3	Nitrate leaching	0.3	kg 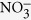 N ha^−1^ yr^−1^ kg^−1^N	33
4	NH_3_-N	0.1	kg NH_3_-N ha^−1^ yr^−1^ kg^−1^ N	33
5	NOx-N	0.005	kg NOx-N ha^−1^ yr^−1^ kg^−1^ N	34
6	CH_4_-C uptake (Upland soil)	−0.012	kg CH_4_-C ha^−1^ yr^−1^ kg^−1^ N	24
7	CH_4_-C emission (Lowland soil)	0.008	kg CH_4_-C ha^−1^ yr^−1^ kg^−1^ N	24
8	CO_2_-C uptake	−0.053	kg CO_2_-C ha^−1^ yr^−1^ kg^−1^ N	24

**Table 4:** Global temperature change potential (kg CO_2_ kg^−1^ N) of different species used in this study.

Species	GTP_20_	GTP_100_	Source
N_2_O	+260 to +290	+290 to +320	13
NOx → ozone and CH_4_	−55 to −37	−2.9 to −0.024	35
NOx → aerosol	−31 to −7	−0.0024 to 0	36
NH_3_ → aerosol	−9.5 to −2.2	−0.022 to 0	36
N fertilizer → CH_4_ flux	+37 to +77	+2.9 to +4.9	37
N fertilizer → CO_2_ flux	+1	+1	38
